# Time trends in smoking in Russia in the light of recent tobacco control measures: synthesis of evidence from multiple sources

**DOI:** 10.1186/s12889-020-08464-4

**Published:** 2020-03-23

**Authors:** Vladimir M. Shkolnikov, Elena Churilova, Dmitry A. Jdanov, Svetlana A. Shalnova, Odd Nilssen, Alexander Kudryavtsev, Sarah Cook, Sofia Malyutina, Martin McKee, David A. Leon

**Affiliations:** 1 Max Planck Institute for Demographic Research, Konrad-Zuse-Str. 1, 18057 Rostock, Germany; 2 National Research University Higher School of Economics, Bolshoy Trekhsvyatitelsiy pereulok 3, Moscow, Russian Federation 109038; 3 National Medical Research Centre for Preventive Medicine, Petroverigskiy pereulok 10, Moscow, Russian Federation 101990; 4 UiT the Arctic University of Norway, 9037 Tromsø, Norway; 5 Northern State Medical University, Troitskiy Avenue 51, Arkhangelsk, Russian Federation 163000; 6 London School of Hygiene & Tropical Medicine, WC1E 7HT, London, UK; 7 Institute of Internal and Preventive Medicine, Siberian Branch of the Russian Academy of Sciences, Vladimirovsky spusk 2a, Novosibirsk, Russian Federation 630003

**Keywords:** Tobacco epidemic, Anti-smoking measures, Male-female gap, Educational differences, Forest plots

## Abstract

**Background:**

The study aims at identifying long-term trends and patterns of current smoking by age, gender, and education in Russia, including the most recent period from 2008 during which tobacco control policies were implemented, and to estimate the impact on mortality of any reductions in prevalence. We present an in-depth analysis based on an unprecedentedly large array of survey data.

**Methods:**

We examined pooled micro-data on smoking from 17 rounds of the Russian Longitudinal Monitoring Study of 1996–2016, 11 other surveys conducted in Russia in 1975–2017, and two comparator surveys from England and the USA. Standardization by age and education, regression and meta-analysis were used to estimate trends in the prevalence of current smoking by gender, age, and educational patterns.

**Results:**

From the mid-1970s to the mid-2000s smoking prevalence among men was relatively stable at around 60%, after which time prevalence declined in every age and educational group. Among women, trends in smoking were more heterogeneous. Prevalence more than doubled above the age of 55 years from very low levels (< 5%). At younger ages, there were steep increases until the mid-2000s after which prevalence has declined. Trends differed by educational level, with women in the lowest educational category accounting for most of the long-term increase. We estimate that the decline in male smoking may have contributed 6.2% of the observed reduction in cardiovascular deaths among men in the period 2008–16.

**Conclusions:**

The implementation of an effective tobacco control strategy in Russia starting in 2008 coincided with a decline in smoking prevalence among men from what had been stable, high levels over many decades regardless of age and education. Among women, the declines have been more uneven, with young women showing recent downturns, while the smoking prevalence in middle age has increased, particularly among those with minimal education. Among men, these positive changes will have made a small contribution to the reduction in mortality seen in Russia since 2005.

## Background

The prevalence of smoking among Russian men has been very high for many years. The WHO Global Adult Tobacco Survey (GATS) found that, in the 2000s, it was among the highest in the world [1] with Russia having the world’s second-largest tobacco market by volume of sales in 2014, [2] even though it is home to under 2% of the world’s population. Encouragingly, over the past 10 years, there have been a series of policy initiatives to tackle this major public health problem. Later than most other countries, in 2008 Russia ratified the Framework Convention on Tobacco Control [3]. From this point on there has been an acceleration in policies aimed at reducing smoking. In 2010 the National Strategy on Creation of a Public Policy to Combat Tobacco Consumption was launched and in 2012 the Ministry of Health issued a decree to introduce pictorial health warnings on cigarette packages from 2013. Most importantly, in 2013 a comprehensive Federal Tobacco Control Law came into force [4]. This included a 100% smoke-free policy in all public places, with the World Health Organisation (WHO) giving Russia a score of 7 on a scale of zero to 10 for compliance, a continued incremental increase in the tax on tobacco products, restrictions on tobacco advertisements (with the WHO scoring Russia as 10 out of 10 for compliance), promotions, and sponsorship (scoring 8–10 for compliance in most categories), and strengthened anti-tobacco campaigns in various media outlets [5].

The implementation of these smoking control policies in Russia overlapped with the longest period of decline in adult mortality since the 1960s. After a period of rising life expectancy after the second world war, the mid-1980s saw the start of massive fluctuations driven mainly by changes in the prevalence of heavy drinking [6]. However, since the mid-2000s, life expectancy has steadily increased, due in particular to declines in mortality from cardiovascular disease (CVD) and external causes. The reasons for this improvement are not fully understood and cannot be attributed solely to changes in alcohol drinking [7, 8]. An important question, therefore, arises as to whether the development of tobacco control policies since 2008 has contributed to these important declines in mortality. However, before this question can be answered we need to know whether smoking prevalence has declined as might be hoped given the policies introduced.

Monitoring smoking behavior in a population is an important public health function. Several countries conduct regular national health and risk-factor monitoring surveys. They include the National Health and Nutrition Examination Survey (NHANES) in the United States and the Health Survey for England (HSE). Russia does not have an equivalent regular population-based public health monitoring survey. Nevertheless, there have been various attempts to measure smoking in Russia, but these have been based mainly on research studies. These include the Russian Longitudinal Monitoring Survey (RLMS) [9, 10], and several other nationally representative surveys [11–14]. Beyond this, there are a number of geographically specific epidemiological studies that have collected data on smoking, even if they have not published detailed analyses of prevalence rates.

Given the importance of looking at whether the recent development of tobacco control policies in Russia has accompanied reductions in smoking, as well as the central role of smoking as a driver of population health, there is a strong argument to undertake a synthesis of all available data sources. Triangulation, whereby one attempts to strengthen the validity of conclusions and inferences, by identifying common patterns from a range of data generated in different ways, is an obvious approach to apply to smoking in Russia.

In this paper, we aim to produce the most in-depth and comprehensive assessment to date of recent trends in current smoking levels for Russia. We consider whether there are different trends by age, sex, and educational level, to inform priorities for future policy. Finally, we consider how far changes in smoking may have contributed to declines in mortality in Russia that have occurred since the mid-2000s.

## Methods

We used our knowledge of the research literature to identify potentially useful data sets. We obtained anonymized individual-level data on smoking from studies of the Russian population. Our criterion for inclusion was that the data came from surveys that were designed with the aim of being representative of the general population of the whole country or of certain cities or regions, including those involving multi-stage sampling with primary sampling units being settlements across the country. Some of the studies, to a greater or lesser extent, are likely to be subject to selection bias and will not be perfectly representative of their target populations. However, by combining data from a range of different studies, using a variety of sampling frames and data collection methods, our logic was that any common patterns that might emerge are likely to reflect the true prevalence of smoking.

### Data sets

In our analysis, we included 17 annual rounds (1996–2016) of the Russian Longitudinal Monitoring Survey (RLMS) plus 11 independent cross-sectional studies conducted between 1975 and 2016. RLMS provides the only continuous time series of smoking in Russia. The RLMS has a design aimed at producing a nationally representative sample (average sample size is 7302 in 1996–2009 and 11,634 in 2010–2016). However, there are some concerns about the continuity of the RLMS time series due to periodic sample replenishments [15]. Replenishment of the 2010 sample was especially significant due to an immediate 50% enlargement of the sample. In the following years, the observed between-round attrition rates were substantially higher than those in 2010 and preceding years [15]. Preliminary checks on the data showed that this temporary distortion also expresses itself in short-term fluctuations in the prevalence of smoking in age groups between 25 and 55. For this reason, RLMS rounds of 2011 and 2012 were excluded from the analysis.

Of the other cross-sectional surveys some have aimed at national representativeness and coverage. These include the Living conditions, Lifestyle and Health (LLH) [12–14] and Health in Times of Transition (HiTT) [14] surveys, the WHO Study on global AGEing and adult health (SAGE), the WHO Global Adult Tobacco Survey (GATS), and the VCIOM “Healthy lifestyle monitoring”. In addition, we have used other ad hoc epidemiological studies that have collected data on smoking, although these are generally restricted to specific geographic locations i.e. cities. The sample size of these varies from 1109 in Izhevsk Family Study 2 (IFS 2) to 73,548 in the Monitoring of Arterial Hypertension (Monitoring AH). While a majority of surveys cover almost the entire range of adult ages from 18 to about 80, the Stress Aging and Health Study (SAHR) and SAGE focus on older ages. The Izhevsk studies IFS 1 and IFS 2 include only those of working age, from 25 to 60. To put our results in an international context, we obtained individual-level data from the 2012 Health Survey for England (HSE) and from the 2015-2016 US National Health and Nutrition Examination Survey (NHANES). More details of the studies included are provided in Table S1 (Additional file 1).

We are aware of several other studies that could have usefully been included. However, despite approaching the data holders for access we were unable to obtain individual-level data. These are the surveys of the epidemiology of cardiovascular diseases and their risk factors in the regions of the Russian Federation (ESSE-RF) funded by the Russian Ministry of Health, [16] the Pitkäranta study in the Republic of Karelia [17] and the Russian component of the HAPIEE study [18].

### Smoking questions

The definition of current smoking varies to some degree across the surveys included in this study (Table S2, Additional file 2). In summary three versions of the current smoking variable were employed: (1) current smoking as defined by the WHO; (2) daily smoking according to the WHO; (3) current smoking based on response to a simple question as to whether they “currently smoked”. According to the WHO definition, current smoking is the consumption of any tobacco product either daily or less frequently (occasionally) (see also Notes under Table S2 in Additional file 2). This broad definition was used in four Russian surveys and in NHANES. In the latter U.S. study, the WHO smoking question was asked only to those who had smoked at least 100 cigarettes during their entire lives. Our cross-tabulation of NHANES data (analysis not shown here) indicates that the 100-cigarette filtering leads to only a 0.9% understatement of current smoking compared to the WHO definition. All RLMS rounds and other five surveys used the conventional question *“Do you smoke in the present?*” (or *“Are you a current smoker*?”). Some of those responding to this question may have assumed that an affirmative answer corresponds to regular daily smoking but others might have thought that it also included periodic or episodic smoking. Our cross-tabulations from GATS, SAGE, and VCIOM surveys suggest that the share of smokers who do not smoke every day constitutes 2 to 7% among those who are current smokers according to the WHO definition. The latter figures reflect a) the magnitude of understatement of the current smoking in surveys where daily smoking is available; and b) the upper limit of the magnitude of understatement of smoking in RLMS and the other five surveys that used the conventional question. These values agree well with the earlier study that assessed the difference in the smoking prevalence between RLMS and GATS [7].

In HSE, the current smoking variable is based on responses to the question “Do you smoke any cigarettes at all nowadays?”. Because of the use of “any” and “at all” this is essentially equivalent to the WHO definition of current smoking.

It is important to note that all the RLMS rounds use the same definition of smoking and in this regard, the RLMS series is fully consistent.

### Statistical analysis

In our main analyses, we adjusted the prevalence of current smoking for educational level by direct standardization. The RLMS educational structure between 1996 and 1998 was taken as a standard for adjustment by education. This was in order to remove the confounding effects of different educational compositions of the various samples both over time and geographically. As education, is very likely to be associated with smoking, the education-adjusted estimates exclude effects that are simply the result of changes or differences in the educational structure of populations. We examined the age-standardized prevalence of current smoking, using the European standard population of 1976 which ensures numerical comparability with prevalence estimates provided by the WHO “Health for All” Database [19].

We then turned to the relationship between smoking and socio-demographic categories over calendar time, firstly across RLMS rounds and then across the other cross-sectional surveys, paying particular attention to whether the time trends were the same. This was done using meta-analytic approaches and where necessary meta-regressions. This approach anticipates and explicitly addresses inter-survey variability of designs and the effects of these on the heterogeneity of estimates.

Multi-study-based relationships between current smoking and three levels of education (lower, secondary, tertiary) were established for each sex separately by running the two-stage individual-participant data meta-analysis (*ipdmetan*) with the forest plot option in Stata 13 [20]. The method first fits a logistic regression linking the odds of smoking with education within each study and then estimates the pooled effect of education as a weighted average of study-specific effects. We used a random-effects model that assumed inverse-variance weights accounting for within-study variance and between-study variance with DerSimonian-Laird estimator [21]. The *I*^2^ heterogeneity measure expresses the between-study variance as a percentage of the total variance. All these estimates (study-specific and pooled effects, effects’ confidence limits, variances and *I*^2^ values) are shown in sex-specific forest plots created using the *forestplot* package in R [22]. To check temporal changes in the age-adjusted prevalence of current smoking for trends, we used the conventional random-effects meta-regression (*metareg*) command in Stata [23].

We assessed a hypothetical impact of the reduction in male smoking on deaths from CVD in 2008–2016. The estimation procedure was based on data that have been used in our earlier estimation of the risk of CVD death in Russia associated with current smoking [24]. We used a proportional hazard model to calculate separate hazard ratios for the three ranges of ages at baseline: 15 to 49, 50 to 64, and 65 and older. We also modeled the time profile of the decline in the excessive risk of CVD death after the elimination of smoking [25]. The calculation method is described in detail in Appendix S1 (Additional file 3).

## Results

Table 1 shows the age and education adjusted prevalence of current smoking by survey year for men and women for each study. We excluded from this table those studies that had a narrow age range (IFS1 and IFS2, SAGE, SAHR), as a statistical adjustment for age would not yield comparable estimates. As the RLMS is an annual panel study using broadly consistent methodology throughout, estimates are shown in a separate column. Overall there is consistency in the trends seen between RLMS and the other studies. Over most of the period studied the age and educational adjusted prevalence of current smoking among men in the Russian studies was relatively stable at around 60% (+/− 3%). From 2009 to 10 there was a suggestion of a decline, with only GATS being an outlier. Importantly, the remarkable reduction to 48% shown in RLMS in 2016 is supported by the findings from two other studies, one of which (VCIOM) had national coverage like RLMS. However, it is notable that the male prevalence even in this most recent year in Russia was much higher than seen in the comparator studies from the UK and USA.

**Table 1:** Age-and-education standardized prevalence of current smoking in RLMS rounds and ad hoc surveys ^a^

Central year	Study	Males (%)		Females (%)	
		Ad hoc	RLMS	Ad hoc	RLMS
1985	LRC/MONICA (1975–2002)	58.2 (1.3)^b^		18.4 (0.9)	
1996	RLMS		62.5(2.8)		12.1 (0.7)
1998	RLMS		60.8 (2.8)		12.3 (0.7)
2000	RLMS		61.0 (2.9)		14.1 (0.8)
2000	Arkhangelsk study (2000)	56.2 (3.2)		22.6 (1.3)	
2001	RLMS		61.9 (2.9)		15.4 (0.8)
2001	LLH (2001)	63.0 (3.8)		17.9 (0.6)	
2002	RLMS		63.4 (2.9)		16.6 (0.9)
2003	RLMS		61.9 (2.9)		16.2 (0.9)
2004	RLMS		62.0 (2.9)		18.0 (1.0)
2005	RLMS		61.6 (3.0)		17.6 (1.0)
2006	RLMS		62.5 (2.6)		18.7 (0.9)
2006	Monitoring AH (2003–2010)	52.3 (0.8)		8.7 (0.1)	
2007	RLMS		62.9 (2.7)		18.8 (0.9)
2008	RLMS		60.6 (2.7)		18.7 (0.9)
2009	RLMS		58.8 (2.6)		17.8 (0.9)
2009	GATS (2009)	62.5 (2.0)		22.8 (0.9)	
2010	RLMS		56.5 (1.9)		17.9 (0.6)
2010	HITT (2010–2011)	56.6 (4.1)		23.5 (1.5)	
2013^c^	RLMS		53.3 (1.8)		17.5 (0.6)
2014	RLMS		52.3 (1.8)		17.8 (0.6)
2015	RLMS		48.6 (1.5)		17.3 (0.7)
2016	RLMS		48.1 (1.9)		17.5 (0.7)
2016	KYH (2015–2017)	48.6 (1.6)		22.2 (0.9)	
2016	VCIOM (2016–2017)	48.7 (2.2)		21.3 (1.4)	
2016	NHANES (2015–2016)	24.2 (1.1)		15.4 (0.7)	
2012	HSE (2012)	23.4 (1.1)		19.2 (0.7)	

*Abbreviations: LRC/MONICA* Lipid Research Clinics and MONItoring trends and determinants in CArdiovascular disease, *LLH* Living conditions, Lifestyle, and Health, *IFS 1 and IFS 2* Izhevsk Family Studies 1 and 2, *Monitoring AH* Monitoring of Arterial Hypertension in the Russian Federation, *SAHR* Stress Aging and Health in Russia, *SAGE* WHO Study on global AGEing and adult health, *GATS* WHO Global Adult Tobacco Survey, *HiTT* Health in Times of Transition, *KYH* Know Your Heart study, *VCIOM* “Healthy lifestyle monitoring” of the All-Russia Center for Studying Public Opinion, *RLMS* Russian Longitudinal Monitoring Survey, *NHANES* National Health And Nutrition Examination Survey, *HSE* Health Survey for England

^a^Four surveys that include only younger ages under 60 (IFS1 and IFS2) and only older ages above 50–55 (SAGE and SAHR) are not shown in this table. They are included in the analyses of age-specific data and in the regression models

^b^Standard errors are given in parentheses

^c^RLMS rounds of 2011 and 2012 are excluded from our analyses for a reason explained in the text (section "Data sets" in the "Methods")

The prevalence of current smoking among women in Russia every year was appreciably lower than in men (Table 1). Across the entire period shown in the RLMS data, there has been a steady increase from around 11% to a high of 19% in 2014. The estimates from the ad hoc surveys for women are generally higher than from the RLMS, with the exception of the Monitoring AH study, again with a suggestion of slightly higher levels after 2009/10. The most recent Russian studies show a prevalence in women that is very similar to that seen in the comparator studies from the UK and the USA.

**Fig. 1:**
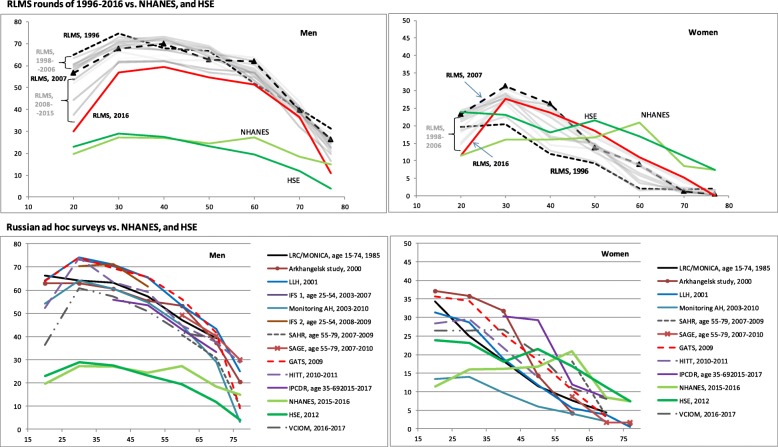
Age-specific prevalence of current smoking adjusted for education in Russian and international surveys

Figure 1 shows the relationship of age to smoking prevalence for men and women separately in different rounds of the RLMS (upper panels) and in the various other studies (lower panels) adjusted for education. In men (left panels) there is a steep increase from adolescence that peaks around the age of 25 years, with a subsequent decline across midlife ages that becomes particularly steep above the age of 60 years. This pattern is almost identical across all the Russian studies, even though there is a downward shift in the prevalence curves for the studies conducted in the most recent years. Other than among the oldest, the prevalence in Russian men is far higher at every age than that in the comparator studies from the UK and the U.S.

Among women Fig. 1 (right panels) also shows a peak prevalence in the early 20s followed by a decline. In the RLMS the prevalence above age 70 is almost zero with the exception of the most recent round in 2016. In Fig. 1 women show a much more diverse range of prevalence values across studies compared to men. Above the age of 55 years among women, the comparator estimates from the USA and UK are higher than at any RLMS round or another Russian study. At younger ages below 35 years the comparator studies, particularly NHANES, show lower prevalence than seen at any point among Russian women.

Figure 1 also shows in more detail the way that smoking prevalence at different age groups has changed over time. For men, it is clear that the lowest prevalence at almost every age was for the most recent year (2016). In contrast, the lowest levels for women in RLMS were seen for the earliest round (1996). For women, it is evident that at younger ages the peak prevalence was in 2007, with prevalences under the age of 45 years falling since then. The relatively complex changes over time seen in the RLMS are not so easy to discern for the other studies (lower panels of Fig. 1), although it is notable that for men the two studies with some of the lowest prevalences at each age are VCIOM and KYH, both of which relate to the most recent period 2015–17. Time trends by age group for RLMS are shown in Figure S1 (Additional file 4).

**Fig. 2:**
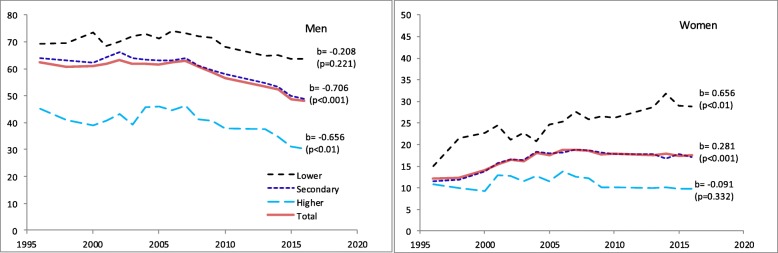
Age-standardized prevalence of smoking in RLMS by education and for the total population in 1996–2016. Note. Values and significance levels for the linear trends’ slopes from meta-regression are shown on the panels

**Fig. 3:**
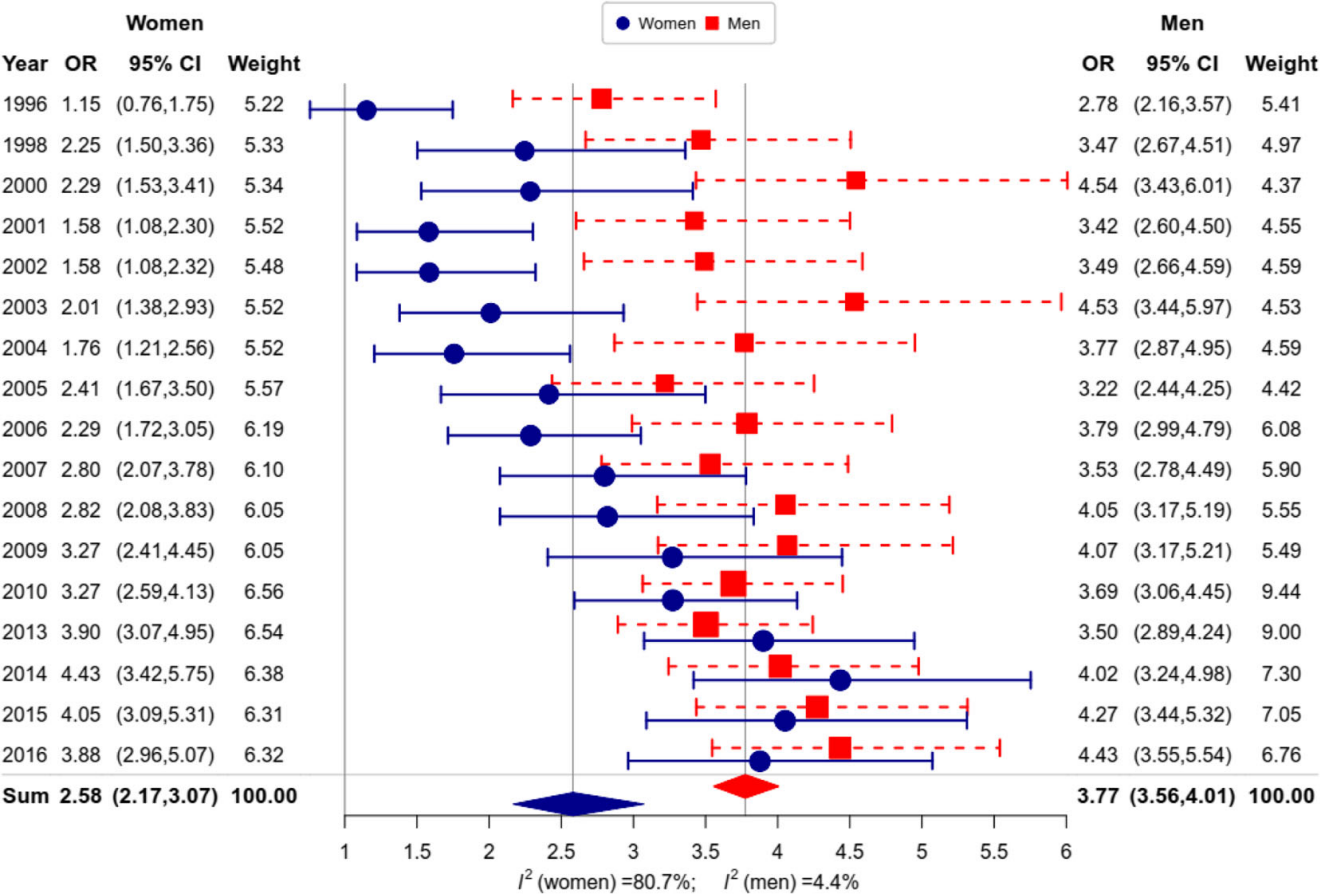
Educational differences in smoking (ORs low vs. high) adjusted for age in RLMS, 1996–2016

We now turn to look at the impact of education on current smoking. The age-standardized prevalence of current smoking by year and education in RLMS is shown in Fig. 2 for men and women separately. For men in all education groups, there has been a decline in prevalence after 2007. The visual impression is confirmed by statistically significant negative meta-regression coefficients. For women, there is no evidence of a decline in any educational group. On the contrary, in the secondary and lower groups, there have been increases, with these being throughout the whole period for the lower group. Because of these different trends, it is clear that over time there has been the emergence of a considerable educational gradient in current smoking among women in the whole period since the mid-1990s. This is demonstrated in Fig. 3, where the odds ratios for being a current smoker in low (vs high) educational group are shown by survey round. For women, there has been a substantial increase in ORs over time from levels 1 to 1.5 in the 1990s to about 3.5 to 4 in the mid-2010s. For men, there is a surprising degree of stability in the ORs at around 3.5 to 4 since 1996. This difference between the two sexes is underlined by the difference in the amount of heterogeneity *I*^2^ which is almost 80% for women, but just 4% for men.

**Fig. 4:**
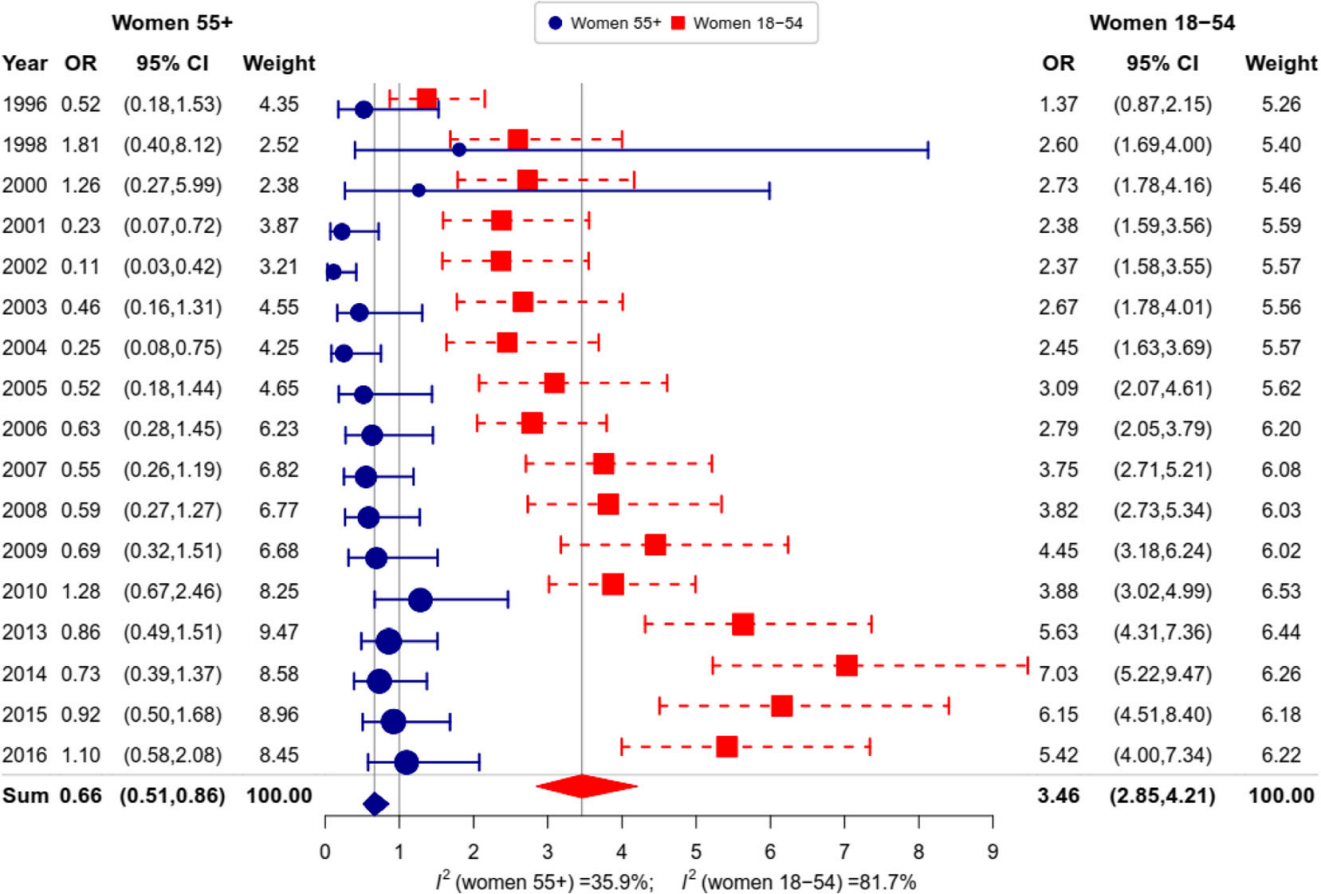
Educational differences in female smoking (ORs low vs. high) by age group in RLMS, 1996–2016

Figure 4 shows that the growing importance of education as a factor of female smoking is entirely driven by smoking at ages under 55. Whereas the OR (higher vs. lower education) remains almost unchanged across RLMS rounds at ages 55+, it sharply increases with time at ages 18 to 54 years.

A parallel analysis looking at the magnitude of the ORs for current smoking by education based on the other surveys is broadly consistent with what we have seen in Figs. 3 and 4 using RLMS. As can be seen in Figure S2 (Additional file 5), the ORs are more stable for men compared to women, where the ORs vary considerably between surveys even when they are conducted in similar periods. In addition, there is only evidence of an educational gradient in female smoking below the age of 55 years (Figure S3 in Additional file 6).

Overall, from our analyses, we have found roughly a 10% decline in the prevalence of current smoking in men in the period 2008–2016. Based on this figure, we estimated the number of male cardiovascular disease (CVD) deaths that might have been avoided because of this positive change. CVD is an appropriate focus because it is the only major group of causes of death where risk rapidly falls to that of non-smokers. We estimate that some 48 thousand CVD deaths were avoided among men compared to a “business as usual” regime in 2008–2016. Of these, 79% would have been under the age of 65 years. This constitutes 1.0% of all deaths during the same nine years under the mortality regime of 2007, 1.3% of all deaths observed in reality in these years, and 6.2% of the reduction in deaths (compared to the mortality regime of 2007) in the observed deaths.

## Discussion

Our novel synthesis of existing data on current smoking prevalence in Russia has shown that for men, the period since 2008 has seen a simultaneous decline in all age groups. This decline is remarkable since it was preceded by a period of many decades where smoking prevalence in men remained almost static at every age. This decline in prevalence has occurred regardless of educational level.

Among women, the increase in smoking prevalence at younger ages appears to have now reversed in the most recent years. However, at older ages, an upward trend remains. These two countervailing trends appear to result in an overall stabilization of the trend for women as a whole. Unfortunately, educational differences in current smoking among women show very divergent trends, with those with the highest levels of education showing small declines, whereas those women with minimal educational attainment have shown a persistent steady increase, resulting in the emergence of a pronounced inverse educational gradient over the last two decades.

Over the past 30 years, smoking in Russia has shown distinctive features that are radically different from those observed in most Western European countries and North America. While smoking prevalence among men has been declining for decades in nearly all high-income countries, in Russia it has been relatively stable. In 2015, WHO projected that male smoking would decline to 54% in 2025 using 3 surveys conducted in the 2000s [26]. Our findings suggest that the decline is underway and that male smoking was below 50% already in 2015–2016. Nevertheless, today the prevalence of current smoking in Russia under the age of 60 years is still far higher than seen in the UK and the USA.

The other distinctive feature is that in the past women in Russia had far lower rates of smoking compared to their counterparts in most Western countries [26]. However, it is known that, since the 1990s, the prevalence of smoking among younger women has been increasing [9]. Our analyses show that compared to current smoking prevalence in the UK and the U.S. up to the age of 45–50, smoking among Russian women is appreciably higher, with the historic low rates remaining confined to older generations.

It is not possible in this analysis to formally evaluate how far the changes in smoking behavior we have found are attributable to the major policy innovations in smoking control introduced in Russia over the past 10 years. A change in smoking prevalence in a population represents the combined effects of three factors, initiation, quitting, and selective mortality. In the absence of long-term cohort data, it is impossible to determine with confidence the precise role that each of the three factors plays. Nevertheless, a substantial decrease both in male and female smoking around age 20 between 2007 and 2016, suggests a lower rate of initiation in younger cohorts (Fig. 1, upper panels). Considerable decreases in smoking of men at ages 30 to 50 and women at ages 30 to 40 may be associated with quitting. These changes in initiation and quitting may have been catalyzed by the policy changes. On the other hand, the rise of smoking among older women may reflect the counter-balancing forces of aggressive marketing by the transnational tobacco industries who saw in the 1990s an unexploited market among Russian women among whom smoking had previously been socially stigmatized [27].

Selective mortality may provide at least a partial explanation of the very pronounced decline in the prevalence of current smoking across ages among males that we have seen. The long-term hazard of death among smokers is about twice that of non-smokers. A simple model we have built (not shown here) suggests that the lower survival of smokers could produce a reduction in the observed cross-sectional prevalence in Russia between ages 25 and 75 of about one quarter. This selective attrition of smokers after age 60 years is particularly pronounced, with half of the rapid decrease in male smoking with age being explained by the high mortality of smokers. This effect would have been particularly pronounced among those who were aged 50 and older in the mid-1990s and who passed through the period of extremely high mortality (“crisis mortality”) in the 1990s and the early 2000s. In contrast among women, because prevalence has been so low, the pattern of smoking from younger to older ages depends mostly on the historical increase in female smoking across generations i.e. cohort increases in smoking.

What is clear is that the policies adopted, focused on price and marketing, are soundly evidence-based and the declines among men have occurred at the same time as their implementation. For younger women, the market downturn in prevalence has also coincided with these smoking control policies being put into effect. To ensure that the tobacco epidemic starts to recede among women overall, in the short and medium-term it will be important to concentrate on measures that encourage people to quit, such as steadily increasing taxes.

In this paper, we have shown that there is consistency across different studies in patterns of smoking behavior among men. This applies to absolute levels, age-patterns, recent declines, and educational gradients. This finding is striking because the studies that were included have a range of designs, sampling frames and were conducted at various points across nearly three decades. This replication of the same pattern provides confidence that in broad terms what we have observed is robust. The stability of patterns seen for men regardless of the survey is notable given the geographic dispersion of the studies. This suggests that there is a geographic homogeneity in the principal patterns of male smoking across the whole of Russia, although the pronounced educational gradients show that smoking among men in the past 20–30 years is not uniform across socio-economic strata.

The situation among women is rather different. We have found a considerably greater heterogeneity in findings across surveys than seen among men. These variations are unlikely to be due to differences in design or sampling frame as the more consistent estimates seen for men are based on the same set of studies. Instead, it is likely to reflect greater geographic variation in smoking behavior among women, consistent with previous analyses that have shown the steepest smoking increases in the large metropolitan centers of Moscow and St Petersburg [9].

In the 1980s and the 1990s, the prevalence of male smoking showed strong educational differences, with the highest levels in the least educated. In comparison among women, educational differences in smoking were initially small but have widened over time. The declines in smoking among men in Russia appear to have occurred in all educational groups. This is the same as has been observed in a diverse range of Western European countries [28] and Canada [29], although unlike in Russia, some other countries have seen the largest declines among the most educated men. In women, the Russian trend of increasing educational differences, driven primarily by increases in prevalence among low educated women is similar to that seen in Western Europe [28] and in Estonia [30], a former Soviet country. Even in the 2010s, age-adjusted smoking did not decrease significantly among Russian women with high education, and it continued to increase in women with low and secondary education.

It has long been assumed that the heavy burden of smoking among men in Russia contributes substantially to the high levels of mortality. For example, using indirect methods, it was estimated that in the year 2000 smoking accounted for 26% of deaths among men. In contrast, the equivalent figure for women was just 3% [31]. What is critical, from a public health perspective, is that individual-level risk of mortality from cardiovascular disease among smokers greatly reduces within a short time after quitting. After 5 years the excessive risk reduces by about 70% and the risk returns to that of non-smokers within 10 years of quitting [25, 32]. This means that declines in smoking prevalence can be expected to have relatively rapid positive effects on the rate of cardiovascular events and deaths in a population. To this extent, the recent decline in smoking among men is already likely to have contributed to the decline in CVD mortality among men in Russia since the late-2000s. Our estimates show that the absolute number of avoided male deaths in 2008–2016 is quite large 48,000, although this constitutes only about 6% of the observed decrease in CVD deaths. Most importantly, maintaining this decline in current smoking will contribute much more to future decreases in CVD, cancers and other diseases. However, the declines in CVD mortality among women that have been seen, particularly at older ages, are almost certainly unrelated to smoking, as trends are in the opposite direction. Moreover, the relatively low prevalence of smoking at older ages in women, where CVD mortality rates are highest, means that smoking among women cannot explain any of the differences in CVD mortality with other countries such as the UK and the USA.

## Conclusion

The implementation of an effective tobacco control strategy in Russia starting in 2008 coincided with a decline in smoking prevalence among men regardless of education from what had been a long-term high and stable level of current smoking. Among women, the declines have been more uneven, with young women showing recent downturns, while the prevalence among those in middle age is increasing, particularly among those with minimal education. Among men, these positive changes will have made a small contribution to the reduction in mortality seen in Russia since 2005.

## Supplementary information

**Additional file 1: Table S1**. Characteristics of studies included in the analysis.

**Additional file 2: Table S2**. Definitions of smoking in surveys.

**Additional file 3: Appendix S1**. Method for estimation of the mortality effect of the reduction of smoking.

**Additional file 4: Figure S1**. Trends in education-standardized prevalence of current smoking by age group in RLMS.

**Additional file 5: Figure S2**. Educational differences in smoking (ORs low vs. high) adjusted for age in various surveys.

**Additional file 6: Figure S3**. Educational differences in female smoking (ORs low vs. high) by age group in various surveys.

## Data Availability

The data that support the findings of this study are available from a variety of different sources (see Table S1 in Additional file 1 for more details). Five of these sources are freely available. Namely, these are: SAGE and GATS surveys of the WHO, 17 rounds of the RLMS of the Higher School of Economics as well as the two international surveys (NHANES of the Centres for Disease Control and Prevention in the USA and HSE of the National Health Service in England). Restrictions apply to the availability of data from the other ten surveys. Data extracts from these surveys were used under license for the current study, and so are not publicly available. The availability of these sources depends on source providers. The authors will facilitate access requests upon the source providers.

## Abbreviations

CVD: Cardiovascular diseases
GATS: WHO Global Adult Tobacco Survey
HiTT: Health in Times of Transition survey
HSE: Health Survey for England.
HAPIEE: Health, Alcohol and Psychosocial factors In Eastern Europe study
IFS 1: Izhevsk Family Study 1
IFS 2: Izhevsk Family Study 2
IPCDR: International Project on Cardiovascular Disease in Russia
KYH: Know Your Heart study
LLH: Living conditions, Lifestyle, and Health survey
LRC/MONICA: Lipid Research Clinics and MONItoring trends and determinants in CArdiovascular disease study
OR: Odds ratio in a logistic regression model
NHANES: National Health And Nutrition Examination Survey
Monitoring AH: Monitoring of Arterial Hypertension in the Russian Federation survey
RLMS: Russian Longitudinal Monitoring Survey
SAHR: Stress Aging and Health in Russia; Lipid Research Clinics/WHO study
SAGE: WHO Study on global AGEing and adult health
VCIOM: “Healthy lifestyle monitoring” of the All-Russia Center for Studying Public Opinion
WHO: the World Health Organization
