# Supplementary material for: Time trends in smoking in Russia in the light of recent tobacco control measures: synthesis of evidence from multiple sources
Source: BMC Public Health. 2020 Mar 23;20:378. doi: 10.1186/s12889-020-08464-4 (PMC7092419; doi:10.1186/s12889-020-08464-4)
Supplement: Supplementary file 1 — Additional file 1: Table S1. Characteristics of studies included in the analysis. [file 12889_2020_8464_MOESM1_ESM.docx]

**Table S1 – Characteristics of studies included in the analysis**

| Study | Full name and reference | Central year | n | Ages | Mean  age | Share  men (%) | Share  lower  and secondary education (%) | Share urban (%) | Type of sample |
| --- | --- | --- | --- | --- | --- | --- | --- | --- | --- |
| LRC/MONICA (1975-2002) | Lipid Research Clinics/WHO MONItoring trends and determinants in CArdiovascular disease [1, 2, 3] | 1985 | 24410 | 18-76 | 46.0 | 74.8 | 58.5 | 100.0 | Seven random population-based samples from two typical districts in Moscow |
| Arkhangelsk study (2000) | Arkhangelsk study [4] | 2000 | 3660 | 18-79 | 42.5 | 53.3 | 78.9 | 100.0 | Random sample of residents registered in one outpatient polyclinic in Arkhangelsk |
| LLH (2001) | Living Conditions, Lifestyle and Health survey [5, 6] | 2001 | 3960 | 18-79 | 45.8 | 43.7 | 78.8 | 72.7 | Nationally representative sample |
| IFS 1  (2003-2007) | Izhevsk Family Study [7] | 2005 | 1920 | 25-54 | 44.6 | 100.0 | 79.9 | 100.0 | Random sample of men living in Izhevsk |
| Monitoring AH  (2003-2010) | Monitoring of Arterial Hypertension [8] | 2006 | 73548 | 18-79 | 45.0 | 39.7 | 81.2 | 70.5 | Random samples of adults from selected  24 Russian regions recruited through outpatient polyclinics |
| IFS 2  (2008-2009) | Izhevsk Family Study 2 [9] | 2008 | 1109 | 27-60 | 48.1 | 100.0 | 79.6 | 100.0 | Follow-up of men surveyed in IFS 1 with additional participants of the same age |
| SAHR  (2007-2010) | Stress Aging and Health in Russia [10] | 2008 | 1619 | 55-79 | 67.0 | 45.9 | 50.5 | 100.0 | Follow-up of a random sample of people aged 55+ from the seven  LRC/MONICA samples |
| SAGE  (2007-2010) | WHO Study on global AGEing and adult health [11] | 2008 | 2809 | 30-79 | 60.5 | 36.2 | 78.7 | 75.2 | Nationally representative sample of people aged 50+ with additional sample of younger people |
| GATS (2009) | WHO Global Adult Tobacco Survey [12] | 2009 | 10875 | 18-79 | 44.8 | 54.7 | 74.1 | 52.6 | Nationally representative sample of people aged 15+ |
| HITT  (2010-2011) | Health in Times of Transition [13, 14] | 2010 | 2911 | 18-79 | 44.5 | 40.5 | 77.5 | 72.5 | Nationally representative sample of people |
| KYH  (2015-2017) | Know Your Heart study[15] | 2016 | 5129 | 35-69 | 53.9 | 42.7 | 60.1 | 100.0 | Random sample of people from Arkhangelsk and Novosibirsk |
| VCIOM  (2016-2017) | "Healthy life style monitoring" of the All-Russia Center for Studying Public Opinion [16] | 2016 | 2615 | 18-79 | 45.4 | 42.1 | 61.1 | 72.5 | Nationally representative sample |
| RLMS rounds of 1996-2016 | Russia Longitudinal Monitoring Survey, 17 rounds [17] | 1996, 1998, 2000,  …,  2016 | 8388 on average | 18-79 | 44.5 in 1996 to 47.5 in 2016 | 42.5 | 79.0 | 66.8 | Nationally representative sample |
| NHANES (2015-2016) | National Health And Nutrition Examination Survey [18] | 2015 | 6118 | 18-79 | 44.4 | 48.4 | - | - | Nationally representative sample |
| HSE (2012) | Health Survey for England [19] |  | 7870 | 18-79 | 47.6 | 48.8 | - | - | Nationally representative sample |

**References**

1. Williams OD, Stinnett S, Chambless LE, Boyle KE, Bachorik PS , Albers JJ, Lippel K. Populations and methods for assessing dyslipoproteinemia and its correlates: the Lipid Research Clinics Program Prevalence Study. Circulation. 1986;73(Supplement I):I4-I11.
2. Deev AD, Konstantinov VV, Shestov DB. Population descriptions and methodology for US-USSR collaboration in Area 1 (Pathogenesis of Atherosclerosis) (Second prevalence study details for Moscow and Leningrad lipid research clinics). Atherosclerosis Reviews. 1988;17:103–9.
3. Deev AD, Oganov RG: Trends and determinants of cardiovascular mortality in the Soviet Union. Int J Epidemiol. 1989;18(Suppl 1):S137-S144.
4. Averina M, Nilssen O, Brenn T, Brox J, Kalinin AG, Arkhipovsky VL. High cardiovascular mortality in Russia cannot be explained by classical risk factors. The Arkhangelsk study 2000. European Journ Epidemiol. 2003;18:871-78.
5. Living conditions, Lifestyles, and Health project. University if Aberdeen. The School of Social Science. <https://www.abdn.ac.uk/socsci/research/new-europe-centre/living-conditions-lifestyles-and-health-project-page-319.php> Accessed 24 October 2018.
6. Leon DA, Saburova L, Tomkins S, Andreev EM, Kiryanov N, McKee M, Shkolnikov VM. Hazardous alcohol drinking and premature mortality in Russia: a population based case-control study. The Lancet. 2007;369(9578):2001-9.
7. Shalnova SA, Balanova YuA, Konstantinov VV, Timofeeva TN, Ivanov VM, Kapustina AV, Deev AD. *Arterialnaya gipertenziya: rasprostranennost‘, osvedomlennost‘, priyom antigipertenzivnikh preparatov i effektivnost‘ lecheniya sredi naseleniya Rossiyskoy Federatcii*.(In Russian). [Artherial hypertension: prevalance, awareness, use of anti-hypertensive drugs, and effectiveness of treatment among population of the Russian Federation]. Russian Journal of Cardiology. 2006;4:45-50.
8. Tomkins S, Collier T, Oralov A, Saburova L, McKee M, Shkolnikov V, Kiryanov N, Leon DA. Hazardous alcohol consumption is a major factor in male premature mortality in a typical Russian city: Prospective cohort study 2003–2009. PLoS One. 2012;7:e30274.
9. Shkolnikova MA, Shalnova SA, Shkolnikov VM, Metelskaya V, Deev AD, Andreev EM, Jdanov D, Vaupel JW. Biological mechanisms of disease and death in Moscow: Rationale and design of the survey on Stress Aging and Health in Russia (SAHR). BMC Public Health. 2009;9:293. <https://www.ncbi.nlm.nih.gov/pmc/articles/PMC2745385/pdf/1471-2458-9-293.pdf> Accessed 24 October 2018.
10. WHO Multi-country studies data archive. Russian Federation - Study on Global Ageing and Adult Health-2007/10, Wave 1. WHO. Report generated on October 24, 2013. <http://apps.who.int/healthinfo/systems/surveydata/index.php/ddibrowser/68/export/?format=pdf&generate=yes>. Accessed 24 October 2018.
11. Global Adult Tobacco Survey (GATS). Russian Federation 2009. Country Report. Russian Ministry opf Health and Social Development, World Health Organisation, U.S. Centers for Disease Control, Research Pulmonology Institute, Rosstat, Global Tobacco Surveillance System, MPOWER, 145 p. Accessed 24 October 2018. <http://www.who.int/tobacco/surveillance/en_tfi_gats_russian_countryreport.pdf>
12. Community Research and Development Information Serice (CORDIS). Projects and Results. HITT-2008. Health in times of transition: trends in population health and health policies in CIS countries. <https://cordis.europa.eu/project/rcn/91038_en.html>. Accessed 24 October 2018.
13. Balabanova D, Roberts B, Richardson E, Haerpfer C, McKee M. Health care reform in the former Soviet Union: Beyond the transition. Health Serv Res. 2012;47(2):840–64.
14. Cook S, Malyutina S, Kudryavtsev AV et al. Know your heart: Rationale, design and conduct of a cross-sectional study of cardiovascular structure, function and risk factors in 4500 men and women aged 35-69 years from two Russian cities, 2015-18. Wellcome Open Res. 2018;3:67. <https://d212y8ha88k086.cloudfront.net/manuscripts/16005/d47d6175-93dd-4c89-b5af-bd17385570f1_14619_-_sarah_cook_v2.pdf?doi=10.12688/wellcomeopenres.14619.2> Accessed 24 October 2018.
15. *Zdoroviy obraz zhizni: monitoring*.(In Russian). [Healthy life style]. VCIOM. <https://wciom.ru/index.php?id=236&uid=116757> .Accessed 24 October 2018.
16. Russia Longitudinal Monitoring Survey – Higher School of Economics (RLMS-HSE). <http://www.cpc.unc.edu/projects/rlms-hse>. Accessed 24 October 2018.
17. National Health and Nutrition Examination Survey. Centres of Disease Control. <https://www.cdc.gov/nchs/nhanes/index.htm>. Accessed 24 October 2018.
18. Health Survey for England - health, social care and lifestyles. National Health Service England. <https://digital.nhs.uk/data-and-information/areas-of-interest/public-health/health-survey-for-england-health-social-care-and-lifestyles>. Accessed October 24 2018.
