# Supplementary material for: Time trends in smoking in Russia in the light of recent tobacco control measures: synthesis of evidence from multiple sources
Source: BMC Public Health. 2020 Mar 23;20:378. doi: 10.1186/s12889-020-08464-4 (PMC7092419; doi:10.1186/s12889-020-08464-4)
Supplement: Supplementary file 2 — Additional file 2: Table S2. Definitions of smoking in surveys. [file 12889_2020_8464_MOESM2_ESM.docx]

**Table S2 – Definitions of smoking in surveys**

| Survey | Definition of smoking |
| --- | --- |
| LRC/MONICA (1975-2002) | Current smoking, conventional |
| Arkhangelsk study (2000) | Current smoking, WHO definition |
| LLH (2001) | Daily smoking, WHO definition |
| IFS 1 (2003-2007) | Current smoking, conventional |
| Monitoring AH (2003-2010) | Current smoking, conventional |
| IFS 2 (2008-2009) | Current smoking, conventional |
| SAHR (2007-2009) | Current smoking, conventional |
| SAGE (2007-2010) | Current smoking, WHO definition |
| GATS (2009) | Current smoking, WHO definition |
| HITT (2010-2011) | Daily smoking, WHO definition |
| KYH (2015-2017) | Current smoking, conventional |
| VCIOM, 2016-2017 | Current smoking, WHO definition |
| RLMS, 17 annual rounds (1994-2016) | Current smoking, conventional |
| NHANES (2015-2016) | Current smoking, WHO definition with additional filtering |
| HSE (2012) | Current smoking, conventional, modified version |

*Notes*
*Current smoking, conventional* - based on the question “Do you smoke in present?” or “Are you a smoker?” or “Are you currently smoking?” or self-definition of respondent’s smoking status as “smoker” or likely formulations.

*Current smoking, WHO definition* - based on the question “Do you currently smoke tobacco on a daily basis, less than daily, or not at all?” This question may be formulated slightly differently in certain surveys (such as SAGE), but it always aims at covering all possible regimes and intensities of tobacco consumption.

*Current smoking in NHANES* – based on the WHO question, but this question is asked to people who had confirmed that they had smoked 100 cigarettes or more during their entire lives.

*Current smoking in HSE* – based on the question “Do you smoke cigarettes *at all* nowadays?”

*Daily smoking, WHO definition* – based on the frequency question “Do you smoke daily or less than daily?”
