# Supplementary material for: Time trends in smoking in Russia in the light of recent tobacco control measures: synthesis of evidence from multiple sources
Source: BMC Public Health. 2020 Mar 23;20:378. doi: 10.1186/s12889-020-08464-4 (PMC7092419; doi:10.1186/s12889-020-08464-4)
Supplement: Supplementary file 3 — Additional file 3: Appendix S1. Method for estimation of the mortality effect of the reduction of smoking. [file 12889_2020_8464_MOESM3_ESM.docx]

**Appendix S1. Method for estimation of the mortality effect of reduction of smoking**

We use a simple and straightforward method for assessment of a hypothetical reduction of smoking on the number of deaths during a fixed period after the reduction begins. We compare absolute numbers of deaths under two mortality regimes: 1) a “business as usual” regime with age-specific death rates remaining the same as in the year preceding the decline in smoking; 2) the smoking reduction regime. The share of smokers at every age is assumed to be diminishing with time as an arithmetic progression with a fixed negative step. The population is assumed to be unchangeable across time. Regime 2) assumes that mortality of initial non-smokers and mortality of smokers remain constant and the relative risk of those quitting diminishes across years of non-smoking according to an *a priori* rule. The difference between the death numbers produced by the mortality regimes 1) and 2) constitutes the avoided deaths.

*Formal framework*

Although the most commonly used CVD risk scores (the European SCORE and the Framingham risk scale) consider age and smoking (as well as other risk factors) as additive (independent) predictors of death from CVD, certain evidence (based on large U.S. sample) suggests that the relative risk associated with smoking varies across ages [1]. In particular, it was shown that the relative risk of CVD death due to smoking tends to be lower at old ages. The latter is important for the assessment of avoided deaths due to the exponential rise of absolute mortality level with age. We take into account variability across ages by expressing relative risk as a function of *x*.

Let us denote $k_{x}(t)$ the inverse of the relative risk of death for smokers at age *x* and time *t*:

$$k_{x}\left( t \right)=\frac{1}{{RR}_{x}(t)}.$$

Assume, that the first annual step of the reduction of smoking takes place between the middle of year 0 and the middle of year 1. At mid-year 0 (just before the smoking begins to decrease) at age *x*, the initial share of smokers is $p_{x}(0)$ and the death rates for smokers and initial non-smokers are $M_{x}^{nsm}$ and $M_{x}^{sm}$, respectively. In year 0 these quantities are:

$M_{x}^{sm}=\frac{M_{x}(0)}{p_{x}\left( 0 \right)+k_{x}\left( 0 \right)-k_{x}(0)p_{x}\left( 0 \right)}$ ,$M_{x}^{nsm}=k_{x}(0)\cdot M_{x}^{sm}$.

Let us assume that the population share of smokers at age *x* (equal to $p_{x}(0)$) after year 0 diminishes annually by the same amount $\Delta_{x}$. Let us assume finally that the relative risk of those, who quit smoking one year ago, two years ago etc. are equal to *RR_x_*(1), *RR_x_*(2) etc. (inverse values *k_x_*(0), *k_x_*(1) etc.). Then the age-specific death rates of the total population at time 0, 1, … , *n* can be expressed as:

year 0: $M_{x}(0)=p_{x}(0)M_{x}^{sm}+(1-p_{x}(0))M_{x}^{sm}k(0)$,

year 1: $M_{x}(1)={(p_{x}(0)-{\Delta_{x})M}_{x}^{sm}+\left( 1-p_{x}\left( 0 \right) \right)M_{x}^{sm}k\left( 0 \right)+\Delta_{x}M_{x}^{sm}k\left( 1 \right)= =M}_{x}\left( 0 \right) -\Delta_{x}M_{x}^{sm}+\Delta_{x}M_{x}^{sm}k\left( 1 \right)$

…..

year *n*: $M_{x}(n)=M_{x}\left( n-1 \right)-\Delta_{x}M_{x}^{sm}+\Delta_{x}M_{x}^{sm}k\left( n-1 \right).$

After *n* years of decline in smoking, the cumulative age-specific absolute number of avoided deaths at age *x* is
${\Delta D}_{x}=n\cdot N_{x}\cdot M_{x}\left( 0 \right)-\sum_{t=1}^{n} (N_{x}\cdot M_{x}\left( t \right))$,
where $N_{x}$ denotes the mid-year population size in year 0 at age *x*.

In the latter equation, the first and the second terms on the right-hand side are equal to the total numbers of deaths that would happen under the “business as usual” conditions and under conditions of decreasing smoking, respectively, in years 1 to *n*. The $\Delta D_{x}$ is the absolute number of avoided deaths. This number can be related to the total number of deaths or to the empirically observed mortality reduction in the years from 1 to n.

In spite of its simplicity and strong assumptions, the described method may be used for approximate evaluation of the magnitude of avoided losses and to see whether these losses are substantial as an absolute number and as a percentage of all deaths and of the empirically observed decrease in deaths over the same time period.

*Calculation*

The method described above was used for the assessment of avoided cardiovascular deaths among men due to a decrease in male smoking in Russia in 2008-2016 (n=9) with 2007 as the initial year (*t*=0). $M_{x}(0)$ values are equal to the age-specific death rates in 2007.

We assume that *RR*_x_(0) is a piecewise constant function of age. According to outcomes of the proportional hazard analysis of a 10-year follow-up of men from the LRC and MONICA studies [2], the *RR_x_*(0) values for CVD mortality risk among male smokers are equal to 2.546, 1.477, and 1.166 for the ranges of ages 15 to 49, 50 to 64, and 65+, respectively.

To determine the *RR*_x_(*t*) (and *k*_x_(*t*)) values, we adopted the model relationship between the risk of CVD death and time after quitting smoking from the supplementary material to the study by Kontis and colleagues [3]. According to the model curve, the excess risk rapidly decreases during the first two years of non-smoking and then decreases at a somewhat slower pace down to null (*RR*_x_=1) in the tenth year. The corresponding *RR*_x_(*t*) values are shown in Table A1.

**Table A1. The relative risk of CVD death across years after the termination of smoking**

| Age | Year0  2007 | Year1  2008 | Year2  2009 | | Year3  2010 | Year4  2011 | Year5  2012 | Year6  2013 | Year7  2014 | Year8  2015 | Year9  2016 | Year10  2017 |
| --- | --- | --- | --- | --- | --- | --- | --- | --- | --- | --- | --- | --- |
| 15-49 | 2.546 | 2.160 | | 1.773 | 1.676 | 1.580 | 1.483 | 1.387 | 1.290 | 1.193 | 1.097 | 1.000 |
| 50-64 | 1.477 | 1.358 | | 1.239 | 1.209 | 1.179 | 1.149 | 1.119 | 1.089 | 1.060 | 1.030 | 1.000 |
| 65+ | 1.166 | 1.125 | | 1.083 | 1.073 | 1.062 | 1.052 | 1.042 | 1.031 | 1.021 | 1.010 | 1.000 |

The number of avoided deaths resulting from the calculation is reported in the main text.

**References**

1. Woloshin S., Schwartz L.M., Welch H.G. The risk of death by age, sex, and smoking status in the United States: putting health risks in context. Journ National Cancer Inst. 2008; 100(12):845-53
2. Jdanov DA, Deev AD, Jasilionis D, Shalnova SA, Shkolnikova MA, Shkolnikov VM. Recalibration of the SCORE risk chart for the Russian population. Eur J Epidemiol. 2014;29(9):621-8.
3. Kontis V, Mathers CD, Rehm J, Stevens GA, Shield KD, Bonita R, et al. Contribution of six risk factors to achieving the 25x25 non-communicable disease mortality reduction target: a modelling study. Lancet. 2014;384(9941):427-37.
