# Supplementary figures and images for: Time trends in smoking in Russia in the light of recent tobacco control measures: synthesis of evidence from multiple sources
Source: BMC Public Health. 2020 Mar 23;20:378. doi: 10.1186/s12889-020-08464-4 (PMC7092419; doi:10.1186/s12889-020-08464-4)

**Figure S1 – Trends in education-standardized prevalence of current smoking by age group in RLMS**

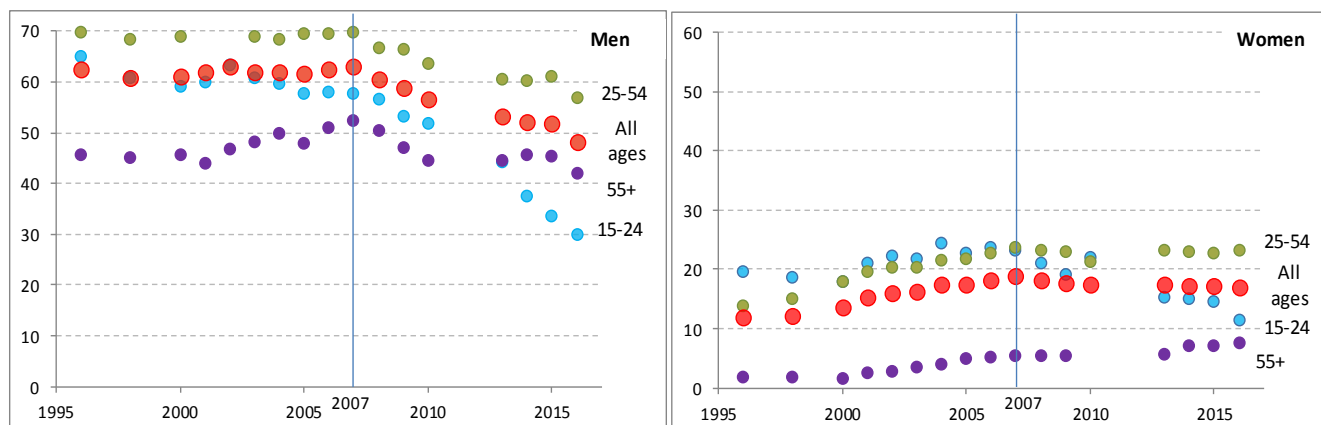

Supplement: Supplementary file 4 — Additional file 4: Figure S1. Trends in education-standardized prevalence of current smoking by age group in RLMS. [file 12889_2020_8464_MOESM4_ESM.pdf]
