# Supplementary material for: Time trends in smoking in Russia in the light of recent tobacco control measures: synthesis of evidence from multiple sources
Source: BMC Public Health. 2020 Mar 23;20:378. doi: 10.1186/s12889-020-08464-4 (PMC7092419; doi:10.1186/s12889-020-08464-4)

Figure S2 - Educational differences in smoking (ORs low vs. high) adjusted for age in various surveys

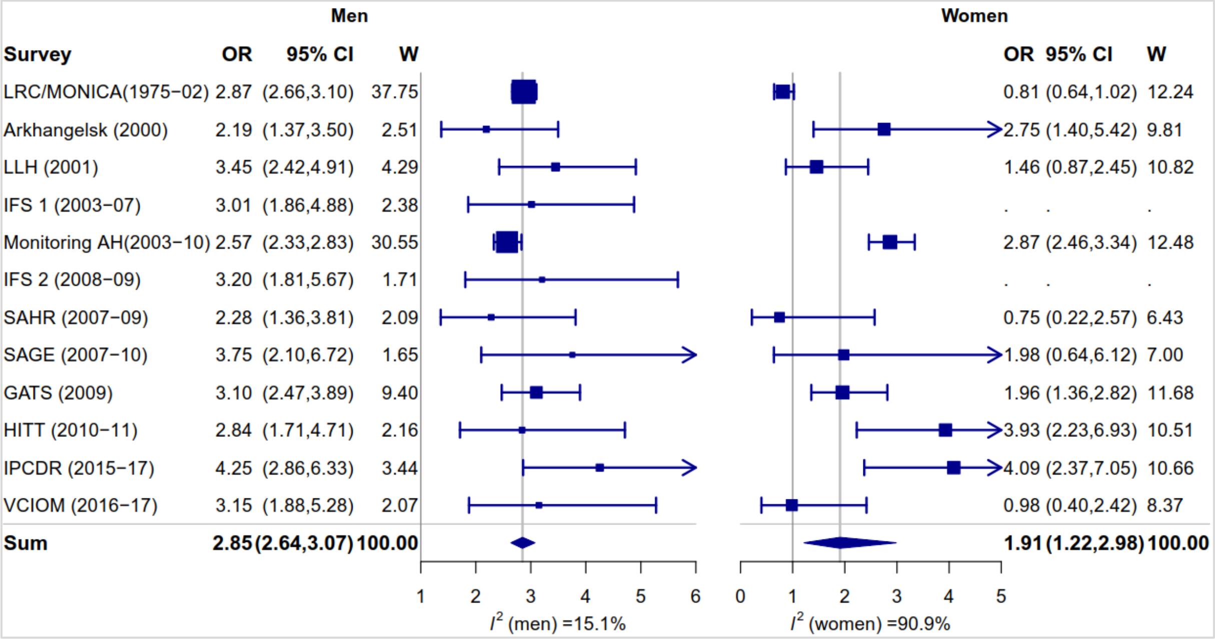

Supplement: Supplementary file 5 — Additional file 5: Figure S2. Educational differences in smoking (ORs low vs. high) adjusted for age in various surveys. [file 12889_2020_8464_MOESM5_ESM.pdf]
